# Nomophobia and Its Associated Factors in Peruvian Medical Students

**DOI:** 10.3390/ijerph19095006

**Published:** 2022-04-20

**Authors:** Cesar Copaja-Corzo, Carlos Jesús Aragón-Ayala, Alvaro Taype-Rondan, Nomotest-Group

**Affiliations:** 1 Facultad de Ciencias de la Salud, Universidad Privada de Tacna, Tacna 23001, Peru; 2 Facultad de Medicina, Universidad Nacional de San Agustín de Arequipa, Arequipa 04001, Peru; caragonay@unsa.edu.pe; 3 Unidad de Investigación para la Generación y Síntesis de Evidencias en Salud, Vicerrectorado de Investigación, Universidad San Ignacio de Loyola, Lima 15001, Peru; alvaro.taype.r@gmail.com

**Keywords:** affective symptoms, addictive, anxiety, internet addiction disorder, nomophobia, Peru, phobic disorders, psychiatric status rating scales, smartphone

## Abstract

Nomophobia is the discomfort caused by not being in contact with a cell phone. Few studies have addressed nomophobia in university students. The study aimed to evaluate nomophobia and its associated factors in Peruvian medical students. We conducted an analytical cross-sectional study on Peruvian medical students between June 2020 and March 2021, using an online survey disseminated through social networks. We analyzed 3139 responses (females: 61.1%, median age: 22 years): 25.7% presented moderate nomophobia and 7.4% severe nomophobia. In the adjusted model, the nomophobia score was lower in students ≥24 years (β: −4.1, 95% CI: −7.2 to −1.0) and was higher in those who had a mobile internet data plan (β: 2.9, 0.8 to 5.0), used the cell phone >4 h (β: 4.5, 2.3 to 6.7), used a smartphone mainly for education (β: 2.5, 0.2 to 4.8), social networks (β: 8.2, 5.8 to 10.6) and entertainment (β: 3.3, 0.5 to 6.1), and those who presented possible anxious (β: 6.6, 4.3 to 8.9) or depressive (β: 19.5, 5.2 to 9.6) symptomatology. In conclusion, nomophobia in university students is a frequent and emerging problem, present mainly at younger ages and associated with symptoms of anxiety or depression. Implementing evaluation and early intervention strategies would favor the mental health of university students.

## 1. Introduction

Smartphones have experienced a rapid expansion worldwide due to their numerous applications, such as internet access, social connectivity, and data storage [1]. In 2019, almost 50% of the world’s population had a smartphone due to its low cost and the ease of communication it provides [2]. People seem to prefer indirect contact and tend to do better in virtual reality, because being behind the screen makes them feel more protected [1]. This shift from real interaction to “social media” has started to negatively impact, for example, addictive behaviors.

Nomophobia (an acronym for “no mobile phone” and phobia) [1] refers to discomfort, anxiety, nervousness, or anguish caused by not having contact with a mobile phone [3]; the term was coined in 2008 by a UK-based research organization [1]. It was found that almost 53% of mobile phone users in Great Britain tend to feel anxious when they “lose their mobile phone, run out of battery or credit or have no network coverage” [1]. Bragazzi et al. [3] have proposed including nomophobia in the Diagnostic and Statistical Manual of Mental Disorders, fifth edition (DSM-V).

Nomophobia is considered a disorder in modern society [1]. It can have various consequences on mental health, affecting work and school performance and generating little social interaction outside of the virtual one [3,4,5,6]. These consequences are significantly negative for medical students and future doctors, who require constant updating of knowledge, which could be diminished in cases of nomophobia [7]. Likewise, in physicians with nomophobia, it could increase the occurrence of medical errors, as has been reported in nursing professionals [5,8,9]. Recent research conducted in different countries [6,8,10,11,12,13,14,15,16,17,18,19] indicates that nomophobia is universally prevalent, with critical geographic differences indicating the need for local studies. In addition, there is little evidence of its possible effects on potentially vulnerable populations such as university students [13,14,16,19].

In this sense, the objective of the study was to evaluate the frequency of nomophobia and its associated factors in medical students in Peru.

## 2. Methodology

### 2.1. Design and Study Environment

We conducted an analytical cross-sectional study on human medical students in Peru. We wrote the manuscript following the guidelines of Strengthening the Reporting of Observational Studies in Epidemiology (STROBE) [20] for reporting observational studies and Checklist for Reporting Results of Internet E-Surveys (CHERRIES) for reporting results of online surveys [21].

### 2.2. Participants

By 2020, Peru had 45 universities with faculties of human medicine [22]. The human medicine career in Peru lasts seven years, with the seventh year dedicated to pre-professional practices and known as the “medical internship”. 

For the present study, we included adult medical students (18 years of age or older) who agreed to participate in the study and stated that they were enrolled in a faculty of human medicine in Peru. We excluded students who did not own a cell phone in the month prior to the survey. We performed convenience sampling, as explained in the following section.

### 2.3. Procedures

After the ethics committee approved the study, we conducted data collection in two stages between June 2020 and March 2021.

First, we created a Facebook page to publicize the research work. We made an open invitation to all human medicine students in Peru using informative posters disseminated in private Facebook groups of human medicine students and through paid Facebook dissemination with selective targeting of medical students in Peru.

In a second stage, through networking in the Peruvian Medical Student Scientific Society, we recruited 23 students from different schools of human medicine as co-investigators. They attended a virtual meeting that lasted 45 min, where they were trained to contact students from their respective universities. To do so, they sent pre-designed private messages through the WhatsApp application, explaining the study, informed consent, and the correct filling out of the survey. If the student agreed to participate in the study, the researchers would send them the link to the online survey in Google Forms. The survey was free for anyone to fill out (no invitation code or other similar mechanism was required). As an incentive, if the student completed the survey, he or she would have access to a Google Drive folder with information collected from open-access medical courses.

### 2.4. Instrument and Variables

We conducted a survey on Google Forms, which we report on.

The survey consisted of 4 sections that were ordered as follows: (1) sociodemographic characteristics (7 questions); (2) smartphone and social network use (4 questions); (3) nomophobia (20 questions); and (4) other scales to assess mental health aspects such as anxiety and depression (25 questions). The survey in its entirety can be found in Supplementary Materials S2.

The questionnaire items were not randomized or tailored (i.e., all respondents answered the same survey, with the questions in the same order). All questions were required to be filled out in Google Forms. We configured the questionnaire only to allow responses after participants provided their email addresses. We did not check that the IP or cookies were not repeated, as we considered that it was possible for different people to respond from the same device.

After choosing the instruments included in the final survey, we conducted a pilot with 30 medical students from the 1st to 6th year (5 students for each year) who were asked to complete the questionnaire. Then, we reported the time they took to fill it out (median time: 10 min) and noted the questions they found ambiguous. With this information, we restructured the order of the instruments and corrected the formulation of the questions.

To assess nomophobia’s severity, we used the Nomophobia Questionnaire (NMP-Q) [23]. This is a self-report questionnaire has 20 items with a 7-point Likert scale score ranging from 1 (“strongly disagree”) to 7 (“strongly agree”), and a total score between 20 and 140 points. In our study, we used the Spanish version of the NMP-Q, validated initially in a population aged 12–19 years in Spain, reporting adequate overall internal consistency (Cronbach’s alpha 0.95), and then used in a similar study in Spain with a population aged 12–24 years [24]. Likewise, we used the following cut-off points for the interpretation of the NMP-Q questionnaire: a score equal to 20 indicates the absence of nomophobia; a score greater than 20 and less than 60 corresponds to a mild level of nomophobia; a score greater than or equal to 60 and less than 100 corresponds to a moderate level of nomophobia; and a score greater than or equal to 100 corresponds to severe nomophobia [23].

To assess anxiety and depression, we used the Hopkins Symptom Checklist-25 (HSCL-25), a shortened version of the HSCL-58 scale [25]. It consists of 25 items (10 to address anxiety and 15 depression). Responses are scored on a 4-point Likert scale, ranging from 1 (“not at all”) to 4 (“very much”). For our study, we used the version validated in Spanish in an adult Peruvian population that presented adequate internal consistency (global Cronbach’s Alpha 0.90; anxiety, α = 0.81; and depression, α = 0.86) [26]. An average score above 1.75 on the anxiety or depression subscales of the HSCL-25 considers that the respondent has possible symptoms of anxiety or depression, respectively [25].

### 2.5. Statistical Analysis

We downloaded the database in a Microsoft Excel document and subsequently exported it for analysis to the statistical program STATA v16. For univariate analysis, we used frequencies, percentages, measures of central tendency, and measures of dispersion.

To evaluate the factors associated with nomophobia, we considered the final NMP-Q score as the dependent variable. We employed linear mixed models to calculate the coefficients (β) and their respective 95% confidence intervals (95% CI). For this, we considered random effects for universities that had more than 50 respondents. As follows: NMP-Q = β0 + β1 ∗ Exposure + … + Zu + e; where: Z = random effect of u, u = university, and e = error. Thus, we calculated the raw and adjusted coefficients (considering all variables with at least one category showing a statistically significant association with the outcome).

For the variable “City where your university is located (categorized according to participants)”, mixed linear models were not used but raw and adjusted because it is highly correlated with the university (which was the variable to which they were assigned random effects).

## 3. Results

### 3.1. Characteristics of the Study Population

During the study period, 3139 human medical students were surveyed, belonging to 38 medical schools located in 18 cities in Peru. The median number of students per medical school was 23 (range: 1–308).

The median age was 22 years, 61.1% were women, 74.6% were economically dependent on their parents, 23.8% were studying at a university located in the city of Lima, and 48.7% were between the fourth and sixth years of their degree.

Regarding the use of cell phones, 76.1% have a mobile internet data plan, 26.7% use the cell phone less than 4 h a day, the social network they used the most was WhatsApp (65.8%). For anxiety and depression symptoms, 34.8% obtained a score greater than or equal to 1.75 (being considered with possible anxiety symptoms), and 42.8% were considered with possible depression symptoms. Regarding the score on the NMP-Q scale, 25.7% presented moderate nomophobia and 7.4% severe nomophobia (Table 1).

### 3.2. NMP-Q Scale Score

Regarding the responses on the NMP-Q scale, the dimensions with the highest average scores were 1 (not being able to access information, mean 2.9) and 4 (not being able to communicate, mean 2.9). The items with the highest mean scores were “I would be upset if I could not consult the information through my smartphone whenever I wanted” (3.4 points). “I would worry about my family and/or friends not being able to contact me” (3.3 points). “If I ran out of data signal or could not connect to a Wi-Fi network, I would be constantly checking to see if I had recovered the signal or managed to find a network” (3.2 points) (Table 2).

### 3.3. Factors Associated with Nomophobia

We created models to evaluate the factors associated with the nomophobia score (numerical variable evaluated with NMP-Q, which could present values between 20 and 140 points).

In the adjusted model, we identified that the nomophobia score was lower in students aged 24 and over (β: −4.1, 95% CI: −7.2 to −1.0) and 21–23 years (β: −3.0, −5.5 to −0.6) (with respect to those aged 18–20 years). Additionally, the score was higher in those who started using their smartphone 7–8 years ago (compared to 0-6 years) (β: 3.9, 1.7 to 6.1), those who had a mobile internet data plan (β: 2.9, 0.8 to 5.0), used the cell phone for 4-5 h (β: 4.5, 2.3 to 6.7), 6–9 h (β: 12.8, 10.3 to 15.4), and for more than 10 h (β: 19.5, 16.0 to 23.0) (compared to those who used it for less than 4 h), who had education (β: 2.5, 0.2 to 4.8), social networking (β: 8.2, 5.8 to 10.6), and entertainment (β: 3.3, 0.5 to 6.1) (compared to those who used it mainly for communication) as their main reason for smartphone use.

In addition to the previous point, the nomophobia score was higher in those students with possible anxiety (β: 6.6, 4.3 to 8.9) and depressive (β: 19.5, 5.2 to 9.6) symptomatology (Table 3).

## 4. Discussion

### 4.1. Prevalence of Nomophobia

We found that approximately one-third of the participating students had moderate or severe nomophobia. This entity could be a proxy indicator of psychiatric comorbidities, such as panic disorders, obsessive-compulsive disorders, eating disorders, and alcohol and drug addictions [3]. In addition, it has been documented that having nomophobia could alter interpersonal relationships [1,4], as well as have a distracting effect on healthcare-related activities [5].

Studies conducted in adolescents and young university adults who also used the NMP-Q instrument and the standard cut-off points [19] showed moderate or severe nomophobia prevalence between 50% and 85% [6,8,10,15,16,17,18]. This prevalence is higher than those reported in our study. Although it is difficult to hypothesize the reasons for this difference, it is possible that it is partly due to the peculiarity of our population. For example, our study was conducted on medical students, who may have a high academic load, leaving them little free time for problematic smartphone use.

It is essential to keep in mind the context of the study conducted between June 2020 and March 2021 during the COVID-19 pandemic. In Peru, a mandatory social quarantine was established from 16 March to 25 October 2020 [27], and from 16 March 2020, all classes and many work activities were conducted virtually, with no return to face-to-face until 2022. This reduced face-to-face interactions and probably generated an increase in the use of smartphones and social networks. However, this could also cause weariness about their use; thus, decreasing the relative importance of some items in the questionnaire.

It should be noted that the cut-off point used for NMP-Q has not been validated in different scenarios, and while some studies find that the main negative consequences of nomophobia occur in moderate to severe cases, others use only the criterion of the highest score [5,28,29] use different cut-off points, or use only some dimensions of nomophobia scale [11,12,14,30,31,32]. This could lead to substantial differences in prevalence reports between studies.

### 4.2. Associated Factors

In this study, we identified that older students had lower nomophobia scores. This is similar to previous studies, which report that higher nomophobia severity may occur in younger populations [1,31,32]. This could be due to the fact that young people are more familiar with technologies and have a higher consumption of social networks [33]. Likewise, we did not find any differences according to sex. Although some studies report higher nomophobia scores in women, this is variable depending on the country of origin and other factors [6,10,13,15,16,30,34,35]. We also report that a higher nomophobia score was associated with the frequency of cell phone use, which is consistent with a study conducted on medical science students in Iran [16].

We identified a positive correlation between nomophobia score and mental health problems (anxiety or depression). This coincides with other studies [1,36,37,38] and may be due to several causes. It is likely that, in people with nomophobia, separation from the cell phone causes worrying thoughts, tense feelings, and even physiological reactions such as sweating, which do not arise in everyday situations [39]. Furthermore, the presence of nomophobia has been associated with the adoption of dysfunctional coping strategies, which may also increase the risk of developing anxiety [40]. On the other hand, other authors have hypothesized that depressed adolescents seek out social networking platforms more aggressively to feel less lonely and good about themselves [36]. Moreover, depressed adolescents’ symptoms are also likely to worsen if they perceive others’ lives to be better than their own through these platforms [31]. A thorough understanding of this association is essential in order to develop appropriate remedial strategies.

### 4.3. Practical Implications

Due to the study’s cross-sectional design, we cannot determine whether mental health disorders would be contributing to nomophobia or vice-versa. However, this association suggests that it may be helpful to look for problems related to nomophobia in students with depression or anxiety. If these are found, we should review the evidence for interventions that may be useful, such as educational and awareness campaigns, the use of applications that focus on reducing screen time on smartphones, therapies such as the cognitive–behavioral approach, or other strategies focused on reducing screen time [41,42], as well as different strategies focused on reducing the levels of nomophobia, such as “mindfulness” or coping strategies to deal with the problem [43,44].

### 4.4. Limitations and Strengths

The present study has certain limitations that should be considered when interpreting the results. Being a cross-sectional study, the direction of the temporality of the associations cannot be assessed. Additionally, being based on self-administered questionnaires, biases such as social desirability bias and recall bias may have been present; however, it was explained to the participants that the survey would be anonymous, which could decrease this risk. In addition, it is likely that some participants may not have understood specific questions, although the researchers were always willing to clarify any doubts. It is also important to mention that specific characteristics and potential confounders of interest, such as alexithymic, impulsive, and metacognitive skills, were not assessed [6]. Finally, it should be considered that participants were approached using Facebook and WhatsApp, so it is likely that users of these applications are over-represented.

Despite the limitations, this is, to our knowledge, the first study that has evaluated nomophobia in university students in Peru. This is a multicenter study that included a large sample and used standardized tools to collect the variables of interest.

## 5. Conclusions

This is, to our knowledge, the first study evaluating nomophobia in university students in Peru. We show that nomophobia is an alarming and emerging problem in the Peruvian context, with a high prevalence among university students, associated with younger ages, prolonged use of the smartphone mainly for purposes other than communication, and possible symptoms of anxiety or depression. Periodic assessment of the status of nomophobia in undergraduate medical students is recommended for further baseline information. Along these lines, it would be essential to include nomophobia and topics related to technological device addiction in the curricula. In addition, it is necessary to evaluate the effects of nomophobia on the performance of physicians working in a clinical setting; thus ensuring patients’ safety by preventing medical errors and addressing the issue from their undergraduate training.

## Figures and Tables

**Table 1:** Characteristics of surveyed students (n = 3139).

Characteristics	n (%)
Sex	
- Male	1220 (38.9)
- Female	1919 (61.1)
Age *	22 (20–24)
How you support your daily expenses	
- Work	174 (5.6)
- Receives money from relatives	2343 (74.6)
- Works and receives money from relatives	622 (19.8)
City where your university is located (categorized by participants):	
- Lima	746 (23.8)
- Arequipa, Trujillo, Chiclayo	615 (19.6)
- Piura, Huancayo, Cusco	813 (25.9)
- Other	965 (30.7)
Year of study	
- 1st to 3rd	1609 (51.3)
- 4th to 6th	1530 (48.7)
Parents’ use of information and communication technologies is defined as “advanced” or “expert”:	
- Father	621 (19.8)
- Mother	393 (12.5)
Years using a Smartphone *	7 (6–9)
Has a mobile internet data plan	
- Yes	2388 (76.1)
- No	751 (23.9)
How much time of the day do you spend using your cell phone?	
- Less than 4 h	838 (26.7)
- 4–5 h	1269 (40.4)
- 6–9 h	758 (24.2)
- 10 h or more	274 (8.7)
The main reason for using your smartphone	
- Communication (video call, SMS, email)	893 (28.5)
- Education	876 (27.9)
- Social networks	858 (27.3)
- Entertainment (gaming, video, music, Netflix)	512 (16.3)
Most used social network	
- WhatsApp	2066 (65.8)
- Facebook	617 (19.7)
- Instagram	324 (10.3)
- Tik Tok	74 (2.4)
- Twitter	57 (1.8)
Nomophobia (NMP-Q)	
- Score (range: 20–140 points) *	47 (33–69)
- No nomophobia (≤20 points)	125 (4.0)
- Mild nomophobia (20–59 points)	1974 (63.0)
- Moderate nomophobia (60–99 points)	807 (25.7)
- Severe nomophobia (100–140 points)	233 (7.3)
Anxiety (HSCL-25)	
- Score (range: 10–40 points) *	15 (12–20)
- Average score ≥ 1.75	1091 (34.8)
Depression (HSCL-25)	
- Score (range: 15–60 points) *	25 (19–32)
- Average score ≥ 1.75	1343 (42.8)

* Median (interquartile range).

**Table 2:** Scores on each item and dimension of the NMP-Q (n = 3139).

Statements	Score (Mean ± Standard Deviation)
**Dimension 1: Not being able to access information**	
- I would be annoyed if I could not consult information through my smartphone whenever I wanted to.	3.4 ± 1.8
- I would be upset if I could not use my smartphone or its apps whenever I wanted to.	3.1 ± 1.8
- I would feel bad if I could not access the information at any time through my smartphone.	3.0 ± 1.8
- I would be nervous if I could not access news (e.g., events, weather forecasts, etc.) via my smartphone.	2.2 ± 1.6
- Average of dimension 1	2.9 ± 1.5
**Dimension 2: Relinquishing comfort**	
- If I were to run out of data signal or be unable to connect to a Wi-Fi network, I would constantly be checking to see if I had regained signal or managed to find a network.	3.2 ± 1.9
- If I could not consult my smartphone for a while, I would feel like doing so.	2.8 ± 1.8
- If I could not use my smartphone, I would be afraid of being stranded somewhere.	2.5 ± 1.8
- I would be scared if my smartphone ran out of battery.	2.4 ± 1.6
- It would give me something if I were about to run out of balance or reach my monthly spending limit.	2.0 ± 1.5
- Average of dimension 2	2.6 ± 1.4
**Dimension 3: Not being able to communicate**	
- I would worry that my family and/or friends would not be able to contact me.	3.3 ± 1.9
- I would worry about not being able to communicate with my family and/or friends at the moment.	3.1 ± 1.8
- I would be anxious about not being able to keep in touch with my family and/or friends.	3.1 ± 1.8
- I would worry about not being in constant contact with my family and/or friends.	2.8 ± 1.8
- I would be nervous about not being able to know if someone has tried to contact me.	2.7 ± 1.8
- I would be nervous about not being able to receive text messages or calls.	2.6 ± 1.8
- Average of dimension 3	2.9 ± 1.6
**Dimension 4: Loss of connection**	
- I would feel uncomfortable not being able to check notifications about my connections and virtual networks.	2.3 ± 1.6
- I would feel strange because I would not know what to do.	2.3 ± 1.7
- I would feel bad for not being able to keep up with what is happening in the media and social networks.	2.2 ± 1.6
- I would be overwhelmed by not being able to check if I have new email messages.	2.2 ± 1.6
- I would be nervous about being disconnected from my virtual identity.	2.1 ± 1.5
- Average of dimension 4	2.2 ± 1.4

**Table 3:** Factors associated with nomophobia (NMP-Q score) in Peruvian medical students (n = 3139).

Feature	The Total Score on the Nomophobia Scale (Mean ± Standard Deviation)	β Crude (95% CI)	β Adjusted (95% CI)
**Sex**			
- Male	52.4 ± 26.6	Ref	
- Female	54.0 ± 26.6	1.3 (−0.6 to 3.2)	
**Age in years**			
- 18–20	57.0 ± 27.1	Ref	Ref
- 21–23	53.3 ± 26.4	**−3.6 (−5.7 to −1.4)**	**−3.0 (−5.5 to −0.6)**
- 24 and over	49.1 ± 25.6	**−7.5 (−9.8 to −5.1)**	**−4.1 (−7.2 to −1.0)**
**How you support your daily expenses**			
- Receives money from relatives	54.4 ± 26.4	Ref	Ref
- Works and receives money from relatives	51.7 ± 26.6	−2.2 (−4.5 to 0.2)	−0.1 (−2.3 to 2.2)
- Work	45.2 ± 27.9	**−8.6 (−12.7 to −4.5)**	−1.6 (−5.7 to 2.4)
City where your university is located (categorized according to participants) *			
- Lima	55.2 ± 26.5	Ref	Ref
- Arequipa, Trujillo, Chiclayo	53.7 ± 25.4	−1.5 (−4.3 to 1.4)	−0.3 (−3.0 to 2.4)
- Piura, Huancayo, Cusco	51.9 ± 26.9	**−3.3 (−5.9 to −0.6)**	−1.2 (−3.7 to 1.3)
- Other	53.1 ± 27.1	−2.0 (−4.6 to 0.5)	1.9 (−0.5 to 4.4)
**Year of study:**			
- 1st to 3rd	55.4 ± 27.2	Ref	Ref
- 4th to 6th	51.3 ± 25.8	**−4.1 (−5.9 to −2.2)**	−1.8 (−4.0 to 0.3)
**Father’s level of “advanced” or “expert” use of information and communication technologies**			
- No	52.8 ± 26.2	Ref	Ref
- Yes	55.8 ± 28.0	**2.4 (0.1 to 4.8)**	−0.6 (−2.9 to 1.8)
**Mother’s “advanced” or “expert” level of information and communication technology use**			
- No	52.9 ± 26.3	Ref	Ref
- Yes	57.0 ± 28.3	**3.8 (1.0 to 6.6)**	2.7 (−0.1 to 5.4)
**How many years ago did you start using a smartphone?**			
- 0–6 years	52.8 ± 26.1	Ref	Ref
- 7–8 years	55.8 ± 27.4	**2.8 (0.6 to 5.1)**	**3.9 (1.7 to 6.1)**
- 9 or more years	51.8 ± 26.3	−1.1 (−3.3 to 1.2)	1.8 (−0.7 to 4.2)
**It has a mobile internet data plan**			
- No	50.2 ± 25.5	Ref	Ref
- Yes	54.4 ± 26.9	**3.8 (1.6 to 6.0)**	**2.9 (0.8 to 5.0)**
**How much time of the day do you use your cell phone**			
- 1–3 h	46.1 ± 24.5	Ref	Ref
- 4–5 h	52.1 ± 25.0	**6.0 (3.8 to 8.3)**	**4.5 (2.3 to 6.7)**
- 6–9 h	59.2 ± 26.7	**12.8 (10.3 to 15.4)**	**9.4 (6.9 to 11.9)**
- 10 h or more	65.7 ± 31.7	**19.5 (16.0 to 23.0)**	**16.5 (13.1 to 19.9)**
**The main reason for using your smartphone**			
- Communication (video call, SMS, mail)	49.0 ± 26.1	Ref	Ref
- Education	51.8 ± 26.1	**2.6 (0.1 to 5.0)**	**2.5 (0.2 to 4.8)**
- Social networks	59.1 ± 27.2	**9.8 (7.3 to 12.3)**	**8.2 (5.8 to 10.6)**
- Entertainment (gaming, video, music, Netflix)	54.1 ± 25.4	**5.0 (2.1 to 7.9)**	**3.3 (0.5 to 6.1)**
**Anxiety (HSCL-25)**			
- Average score < 1.75	48.7 ± 24.9	Ref	Ref
- Average score ≥ 1.75	62.2 ± 27.4	**13.2 (11.3 to 15.1)**	**6.6 (4.3 to 8.9)**
**Depression (HSCL-25)**			
- Average score < 1.75	47.5 ± 25.0	Ref	Ref
- Average score ≥ 1.75	61.2 ± 26.6	**13.4 (11.6 to 15.3)**	**7.4 (5.2 to 9.6)**

Linear mixed models were used, considering the random effects of universities with more than 50 respondents. Statistically significant results are shown in bold. * For the variable “City where your university is located (categorized according to participants)”, linear mixed models were not used because it is highly correlated with the university, but rather crude and adjusted linear regression models.

## Data Availability

Available upon reasonable request.

## Supplementary Materials

The following supporting information can be downloaded at: https://www.mdpi.com/article/10.3390/ijerph19095006/s1. Supplementary Materials S1: Author list of Nomotest-Group; Supplementary Materials S2: Survey used in the study (“NOMOTEST survey”).
