# Supplementary material for: Nomophobia and Its Associated Factors in Peruvian Medical Students
Source: Int J Environ Res Public Health. 2022 Apr 20;19(9):5006. doi: 10.3390/ijerph19095006 (PMC9101167; doi:10.3390/ijerph19095006)
Supplement: Supplementary file 1 [file ijerph-19-05006-s001.zip › ijerph-1660602-supplementary.pdf]

International Journal of
Environmental Research and Public Health

MDPI

Supplemental Material S1: Author list of Nomotest Group (NT-GP)

Scientific name	Affiliation
Cender U. Quispe-Juli	Facultad de Ciencis y Filosofía, Universidad Peruana Cayetano Heredia. Lima, Perú.
Camila I. Medina-Quispe	Universidad Nacional de San Cristobal de Huamanga. Ayacucho, Perú.
Diana M. Ticona	Centro de Investigación de Estudiantes de Medicina Universidad Privada de Tacna, Tacna, Perú.
Noelia Morocho-Alburqueque	Universidad Nacional de Piura. Piura, Perú.
M.G. Alvarez-Valdivia	Facultad de Medicina Humana, Universidad Católica de Santa María, Arequipa, Perú.
Carlos S. Mamani-García	Facultad de Medicina, Universidad Nacional de San Agustín de Arequipa. Arequipa, Perú.
Juan J. Olortegui-Rodriguez	Escuela de Medicina Humana, Universidad Peruana Unión. Lima, Peru.
Veronica Laurel-Vargas	Escuela Profesional de Medicina Humana, Universidad Nacional de San Antonio Abad del Cusco. Cusco, Perú.
Christian Renzo Aquino-Canchari	Facultad de Medicina Humana, Universidad Peruana los Andes. Huancayo, Perú.
Mauricio Herrera-Florez	Facultad de Ciencias de la Salud, Universidad Peruana de Ciencia Aplicadas. Lima, Perú.
Lesly Mendoza-Guillen	Facultad de Medicina San Fernando, Universidad Nacional Mayor de San Marcos (UNMSM). Lima, Perú.
Susan Chaupi-Rojas	Facultad de Medicina Humana. Universidad Ricardo Palma. Lima, Perú.
Akami M. Albitres- Cerna	Sociedad Científica de Estudiantes de Medicina, Universidad Nacional del Santa. Nuevo Chimbote, Perú.
Adai Vera-Luza	Escuela Profesional de Medicina Humana, Universidad Andina del Cusco. Cusco, Perú.
Juan C. Sánchez-Vicente	Facultad de Medicina, Universidad Nacional San Luis Gonzaga. Ica, Perú.
Jhosephi Vasquez-Ascate	Universidad Nacional de la Amazonia Peruana. Iquitos, Perú.

Supplementary Material S2: Survey used in the study ("NOMOTEST survey")

NOMOTEST

a) Answer or mark with a cross (X) the following questions:

1. In what year were you born? _____

2. Sex:

(0) Male

(1) Female

3. What city are you from? _____________

4. How do you support your daily expenses?

(1) Works

(2) Receives money from relatives

(3) Works and receives money from family members

5. About this semester:

5.1 What year are you in?

(1) First year

(2) Second year

(3) Third year

(4) Fourth year

(5) Fifth year

(6) Sixth year

6. About your dad:

6.1 What is your level of use of information and communication technologies (ICT)?

(1) Beginner

(2) Moderate

(3) Advanced

(4) Expert

7. About your mom:

7.1 What is your level of use of information and communication technologies (ICT)?

(1) Beginner

(2) Moderate

(3) Advanced

(4) Expert

8. At what age did you start using a smartphone? ____

9. About your cell phone:

9.1 Do you have a mobile internet data plan?

(1) Yes

(2) No

9.2 How much of your day do you spend using your cell phone?

(1) 1-3 h

(2) 4-5 h

(3) 6-9 h

(4) 10 h or more

9.3 What is the main reason you use your smartphone?

(1) Education

(2) Communication (video call, SMS, email)

(3) Social networks

(4) Entertainment (games, video, music, Netflix)

Do you experience discomfort or discomfort in your legs, combined with the urge or need to move them? (If no, skip questions 15, 16, and 17):

(1) Yes

(2) No

11. Do these discomforts occur only at rest and improve with movement?

(1) Yes

(2) No

12. Are they worse in the evening than in the morning?

(1) Yes

(2) No

13. How often does this happen to you?

(1) Less than one time per year

(2) 1 time or more per year but less than one time per month

(3) 1 time per month

(4) 2-4 times per month

(5) 2-3 times per week

(6) 4-5 times per week

(7) 6-7 times per week

International Journal of
Environmental Research and Public Health

MDPI

b) Answer or mark with a cross (X) the box with the corresponding letter:

	Nothing	A little	Quite	Much
1. Suddenly panics for no reason	a	b	c	d
2. You feel you are afraid	a	b	c	d
3. Fainting, dizziness, or weakness	a	b	c	d
4. You feel nervousness or uneasiness inside you	a	b	c	d
5. Your heart beats fast or faster than usual	a	b	c	d
6. Tremor	a	b	c	d
7. Feeling tense or trapped	a	b	c	d
8. Headaches	a	b	c	d
9. Periods of terror or panic	a	b	c	d
10. You feel uneasy or that you cannot stay calm	a	b	c	d
11. Feeling lack of strength, sluggishness	a	b	c	d
12. Persistent feelings of guilt. Regrets	a	b	c	d
13. Cries easily	a	b	c	d
14. Has lost sexual interest or pleasure	a	b	c	d
15. Lack of appetite	a	b	c	d
16. Difficulty falling asleep or staying asleep	a	b	c	d
17. Feeling hopeless about the future	a	b	c	d
18. Feeling sad	a	b	c	d
19. Feeling lonely	a	b	c	d
20. He has thought about ending his life	a	b	c	d
21. Feeling trapped or imprisoned	a	b	c	d
22. Worries excessively about things	a	b	c	d
23. Lack of interest in things	a	b	c	d
24. Feels that everything requires too much effort	a	b	c	d
25. Feeling useless	a	b	c	d

In the following questions, mark with a cross (x) according to your perception, where: 1 is "strongly disagree," and seven is "strongly agree."

1. I would feel bad if I could not access information at any time through my smartphone.	1	2	3	4	5	6	7
2. I would be annoyed if I could not consult information through my smartphone whenever I wanted to.	1	2	3	4	5	6	7

3. I would be nervous if I could not access news (e.g., events, weather forecasts, etc.) via my smartphone.	1	2	3	4	5	6	7
4. I would be upset if I could not use my smartphone and/or its apps whenever I wanted to.	1	2	3	4	5	6	7
5. I would be scared if my smartphone ran out of battery.	1	2	3	4	5	6	7
6. It would give me something if I were about to run out of balance or reach my monthly spending limit.	1	2	3	4	5	6	7
7. If I were to run out of data signal or be unable to connect to a Wi-Fi network, I would constantly be checking to see if I had regained signal or managed to find a network.	1	2	3	4	5	6	7
8. If I could not use my smartphone, I would be afraid of being stranded somewhere.	1	2	3	4	5	6	7
9. If I could not consult my smartphone for a while, I would feel like doing so.	1	2	3	4	5	6	7
10. I would worry about not being able to communicate with my family and/or friends at the moment.	1	2	3	4	5	6	7
11. I would worry that my family and/or friends would not be able to contact me.	1	2	3	4	5	6	7
12. I would be nervous about not being able to receive text messages or calls.	1	2	3	4	5	6	7
13. I would be uneasy about not being able to keep in touch with my family and/or friends.	1	2	3	4	5	6	7
14. I would be nervous about not being able to know if someone has tried to contact me.	1	2	3	4	5	6	7
15. I would worry that I am no longer in constant contact with my family and/or friends.	1	2	3	4	5	6	7
16. I would be nervous about being disconnected from my virtual identity.	1	2	3	4	5	6	7
17. I would feel bad for not being able to keep up with what is happening in the media and social networks.	1	2	3	4	5	6	7
18. I would feel uncomfortable not being able to check notifications about my connections and virtual networks.	1	2	3	4	5	6	7
19. I would be overwhelmed by not being able to check if I have new email messages.	1	2	3	4	5	6	7
20. I would feel weird because I would not know what to do.	1	2	3	4	5	6	7

The following questions ask how you have slept DURING THE PAST MONTH (4 weeks). Try to answer as accurately as possible what happened during most of the days and nights of the last month:

1. In the last four weeks,

Write your usual bedtime: __: __

2. In the last four weeks, how long did it usually take you to fall asleep (fall asleep) at night?

Write the time in minutes: ___

3. In the last four weeks, at what time did you usually get out of bed in the morning and not go back to sleep? (Use 24 h system)

Write your usual time to get up: __: __

4. In the last four weeks, on average, how many effective hours have you slept per night? (Time may be different from the time you stay in bed)

Write the number of hours you think you slept: ___

	**0. Not at all in the last month**	**Less than once a week**	**2. Once or twice a week**	**3. Three or more times a week**
**5. In the last four weeks, how many times have you had trouble sleeping because of...**				
a. Not being able to fall asleep in the first half-hour?	0	1	2	3
b. Waking up during the night or early morning?	0	1	2	3
c. Having to get up early to go to the bathroom?	0	1	2	3
d. Not being able to breathe well?	0	1	2	3
e. Coughing or snoring noisily?	0	1	2	3
f. To feel cold?	0	1	2	3
g. Feeling too hot?	0	1	2	3
h. Having nightmares or "bad dreams"?	0	1	2	3
i. Suffering from pain?	0	1	2	3
j. Other reasons: ____________________? (Specify)	0	1	2	3
**6. During the last four weeks, how many times have you taken sleeping medication (prescribed by your doctor or on your own)?**	0	1	2	3

**7. During the last four weeks, how many times have you felt drowsy (or very sleepy) while driving, eating, working, studying, or doing any other activity?**	0	1	2	3
	**0. None**	**1. Little**	**2. Fair or Moderate**	**3. A lot or Quite a lot**
**8. During the last four weeks, how much of a problem have you had keeping yourself upbeat or enthusiastic about your tasks or activities?**	0	1	2	3
	**0. Pretty good**	**1. Good**	**2. Bad**	**3. Pretty bad**
**9. During the last four weeks, how would you rate the quality of your sleep?**	0	1	2	3
